# Tacrolimus versus cyclosporine a combined with post-transplantation cyclophosphamide for AML In first complete remission: a study from the acute leukemia working party (EBMT)

**DOI:** 10.1038/s41409-024-02331-1

**Published:** 2024-07-03

**Authors:** Gesine Bug, Myriam Labopin, Alexander Kulagin, Didier Blaise, Anna Maria Raiola, Jan Vydra, Simona Sica, Mi Kwon, Lucía López-Corral, Stefania Bramanti, Peter von dem Borne, Maija Itälä-Remes, Massimo Martino, Yener Koc, Eolia Brissot, Sebastian Giebel, Arnon Nagler, Fabio Ciceri, Mohamad Mohty

**Affiliations:** 1 Goethe University Frankfurt, University Hospital, Dept of Medicine 2, Frankfurt am Main, Germany; 2 Sorbonne University, Department of Hematology, Saint Antoine Hospital, INSERM UMR 938, Paris, France; 3 RM Gorbacheva Research Institute, Pavlov University, St. Petersburg, Russian Federation; 4 Transplant and cellular immunotherapy program, Department of hematology, Institut Paoli Calmettes, Management Sport Cancer lab, Luminy, Aix Marseille University, Marseille, France; 5 IRCCS San Martino Hospital, Genova, Italy; 6 Institute of Hematology and Blood Transfusion, Prague, Czech Republic; 7 Dipartimento di Diagnostica per Immagini, Radioterapia Oncologica ed Ematologia, Fondazione Policlinico Universitario A. Gemelli IRCCS, Università Cattolica Sacro Cuore, Rome, Italy; 8 Department of Hematology, Hospital General Universitario Gregorio Marañon, Instituto de Investigación Sanitaria Gregorio Marañón, Univesidad Complutense de Madrid, Madrid, Spain; 9 Hospital Universitario de Salamanca (Spain), IBSAL, Centro de Investigación del Cáncer-IBMCC (USAL-CSIC), Salamanca, Spain; 10 Department of Oncology/Hematology, IRCCS Humanitas Research Hospital, via Manzoni 56, 20089 Rozzano, Milan, Italy; 11 Leiden University Medical Center, Leiden, Netherlands; 12 Turku University Hospital, Turku, Finland; 13 Stem Cell Transplantation and Cellular Therapies Unit (CTMO), Grande Ospedale Metropolitano “Bianchi-Melacrino-Morelli”, Reggio Calabria, Italy; 14 Medicana International Hospital, Istanbul, Turkey; 15 Maria Sklodowska-Curie National Research Institute of Oncology, Gliwice Branch, Gliwice, Poland; 16 Chaim Sheba Medical Center, Tel Hashomer, Israel; 17 IRCCS San Raffaele Scientific Institute, Milan, Italy

**Keywords:** Acute myeloid leukaemia, Stem-cell therapies

## Abstract

Choice of calcineurin inhibitor may impact the outcome of patients undergoing T-cell replete hematopoietic cell transplantation (HCT) with post-transplant cyclophosphamide (PT-Cy) and mycophenolate mofetil (MMF) for prophylaxis of graft-versus-host disease (GVHD). We retrospectively analyzed 2427 patients with acute myeloid leukemia (AML) in first remission transplanted from a haploidentical (*n* = 1844) or unrelated donor (UD, *n* = 583) using cyclosporine A (CSA, 63%) or tacrolimus (TAC, 37%) and PT-Cy/MMF. In univariate analysis, CSA and TAC groups did not differ in 2-year leukemia-free or overall survival, cumulative incidence (CI) of relapse or non-relapse mortality. CI of severe grade III-IV acute GVHD was lower with TAC (6.6% vs. 9.1%, *p* = 0.02), without difference in grade II-IV acute GVHD or grade III-IV acute GVHD/severe chronic GVHD, relapse-free survival (GRFS). In multivariate analysis, TAC was associated with a lower risk of severe grade III-IV acute GVHD solely with haploidentical donors (HR 0.64 [95% CI, 0.42–0.98], *p* = 0.04), but not UD (HR 0.49 [95% CI, 0.2–1.21], *p* = 0.12). There was no significant difference for chronic GVHD. In conclusion, PT-Cy/MMF-based GVHD prophylaxis resulted in favorable OS and GRFS, irrespective of the CNI added. In haploidentical HCT, TAC seemed to prevent severe acute GVHD more effectively than CSA without impact on other outcome parameters.

## Introduction

Allogeneic hematopoietic cell transplantation (HCT) is the recommended consolidation therapy for the majority of intensively treated patients with acute myeloid leukemia (AML) in first complete remission (CR1), resulting in long-term survival rates of 45–60% [1–3]. For patients who lack a matched related or unrelated donor (UD), HCT from a haploidentical family donor is an alternative. Haploidentical T cell-replete HCT has become feasible by combining a calcineurin inhibitor (CNI) and mycophenolate mofetil (MMF) with high-dose post-transplantation cyclophosphamide (PT-Cy) [4], resulting in survival outcomes similar to human leukocyte antigen (HLA)-matched HCT with conventional graft-versus-host disease (GVHD) prophylaxis using a CNI and methotrexate (MTX) [5, 6]. PT-Cy is highly effective in preventing GVHD by modulating alloreactive T cell responses, i.e. reducing proliferation of CD4 + T cells, depleting activated CD4+ and CD8+ effector T cells and promoting CD4+ regulatory T cell recovery, and thereby promoting tolerance [7, 8].

The original Baltimore protocol for haploidentical HCT combined a non-myeloablative (NMA) conditioning regimen with PT-Cy, the CNI tacrolimus (TAC) and MMF [4]. However, no difference in outcomes between TAC and cyclosporine A (CSA) was observed in a retrospective analysis of NMA conditioning haploidentical HCT in patients with lymphoid malignancies. All but one patient in the TAC group received a bone marrow graft, while peripheral blood stem cells (PBSC) predominated in the CSA group [9]. In patients with acute leukemia in CR1, the feasibility of haploidentical bone marrow transplantation using PT-Cy, CSA and MMF has been demonstrated with a leukemia-free survival (LFS) probability at 5 years of 64% [10].

More recently, PT-Cy has also been adopted as GVHD prophylaxis after UD-HCT, based on two prospective randomized trials reporting superior GVHD-free, relapse-free survival (GRFS) in patients receiving a PT-Cy-containing regimen compared to a CNI combined with MMF or MTX: The HOVON-96 trial evaluated the impact of PT-Cy and a short course of CSA with CSA and mycophenolate acid (MPA), the active form of MMF, on the composite endpoint GRFS in 160 patients with high-risk hematologic malignancies including 30% AML. Patients were mostly treated with an NMA conditioning regimen and grafted from a matched related or UD. PT-Cy/CSA was associated with significantly improved GRFS at one year irrespective of donor type (45% vs. 21%, *p* < 0.001), due to a lower incidence of grade II-IV acute GVHD and extensive chronic GVHD [11]. In the BMT CTN 1703 study, 431 patients (48% AML) were randomized to receive PT-Cy, TAC and MMF, or TAC/MTX after a matched related or UD reduced-intensity conditioning HCT. Again, patients in the PT-Cy group experienced less severe acute or chronic GVHD resulting in a significantly better GRFS at one year (52.7% vs. 34.9%) [12]. In both trials, overall survival (OS), non-relapse mortality (NRM) and relapse incidence did not differ between patients in the PT-Cy/CNI and CSA/MMF or TAC/MTX group, respectively.

To date, it is not known whether the choice of CNI impacts outcomes in patients with AML in CR1 in the setting of PT-Cy-based, T-cell replete HCT. We retrospectively compared GVHD prophylaxis with CSA versus TAC in combination with PT-Cy and MMF in a large homogeneous cohort of patients with AML in CR1.

## Methods

### Data collection

Data for this retrospective multicenter study were retrieved from the registry of the Acute Leukemia Working Party (ALWP) of the European Society for Blood and Marrow Transplantation (EBMT), a nonprofit, scientific society representing >600 transplant centers, mainly located in Europe. Centers commit to reporting all consecutive HCTs and follow-ups once a year. Data are entered, managed, and maintained in a central database and validated by verification of the computer printout of the entered data, cross-checking with the national registries, and on-site visits to selected teams. All patients gave informed consent authorizing the use of their personal information for research purposes. This study was approved by the ALWP of the EBMT institutional review board and conducted per the Declaration of Helsinki and Good Clinical Practice guidelines.

### Criteria for patient selection

Patient selection was based on the following criteria: 1) Adult patients with AML in CR1 who received 2) a first allograft in 2010–2021 using 3) bone marrow or PBSC from a matched or mismatched UD or haploidentical donor and 4) GVHD prophylaxis with PT-Cy, CSA and MMF or PT-Cy, TAC and MMF. Patients undergoing in vivo T-cell depletion or receiving an ex vivo manipulated graft were not included.

### Statistical analysis

The primary endpoint of this study was OS; secondary endpoints included LFS, cumulative incidence (CI) of relapse, NRM, incidence of acute and chronic GVHD, as well as grade III-IV acute GVHD/ severe chronic GVHD, relapse-free survival (GRFS) [13]. Acute and chronic GVHD were diagnosed according to the modified Glucksberg criteria and modified Seattle criteria, respectively [14, 15].

Patient, disease, and transplant characteristics were compared by using the χ^2^ or Fisher’s exact test for categorical variables and the Mann-Whitney test for continuous variables. Probabilities for OS, LFS and GRFS were calculated using Kaplan-Meier estimates, and CI of relapse, NRM, acute and chronic GVHD using a competing risk model: relapse and death are competing risks, i.e. relapse is the competing event for NRM, and death without relapse the competing event for relapse, whereas relapse and death are competing risks for GVHD [16]. Univariate analyses were performed using the log-rank test for LFS, OS, and GRFS, and Gray’s test for CI estimates [17]. A Cox’s proportional hazards model was used for multivariate analyses by including all variables differing significantly between the groups, and factors known to influence outcomes including donor type, age, secondary AML, adverse risk cytogenetics according to the 2017 European LeukemiaNet (ELN) classification, Karnofsky performance status, reduced-intensity versus myeloablative conditioning (MAC) regimen, female donor to male recipient combination, cytomegalovirus seropositive recipient, donor type and graft source. In order to take into account the heterogeneity in the effect of a characteristic or a treatment across centers, we introduced a random effect into Cox multivariate models [18].

All tests were two-sided with the type 1 error rate fixed at 0.05. SPSS 27.0 (IBM Corp., Armonk, NY, USA) and R 4.0.2 (R Core Team 2020. R: a language and environment for statistical computing. R Foundation for Statistical Computing, Vienna, Austria, https://www.Rproject.org/), were used for all statistical analyses.

## Results

### Patients and transplant procedures

This large retrospective study included 2427 AML patients who had received a first unmanipulated HCT from a haploidentical donor (*n* = 1844, 76%) or UD (*n* = 583, 24%) in 2010–2021. GVHD prophylaxis consisted of PT-Cy/MMF/CSA (*n* = 1528, 63%) or PT-Cy/MMF/TAC (*n* = 899, 37%) without in vivo T-cell depletion. Patient characteristics were well balanced in the CSA and TAC groups with respect to age (median 55.1 [range, 18.1–75.6] vs. 54.9 [range, 18–82.5] years), Karnofsky performance score <90 (20.8% vs. 21.8%), and adverse risk cytogenetics (27.1% vs. 26.3%), respectively, as shown in Table 1. Stem cell source and donor type differed between groups, with patients on CSA-based GVHD prophylaxis being more likely to have received bone marrow (31.3% vs. 16.1%) and a graft from a haploidentical donor (81.0% vs. 67.4%), *p* < 0.0001 each. Negative measurable residual disease (MRD) status prior to HCT (62.6% vs. 65.7% of 1164 patients) and use of MAC (49.8% vs. 47.9%) were similar in both cohorts. The conditioning regimen most frequently combined with CSA was thiotepa/busulfan/fludarabine (TBF, 56.3% of patients), followed by fludarabine/busulfan (FluBu, 19.9%) and fludarabine/total body irradiation (FluTBI, 13.5%). TAC was mostly given after FluBu (35.9%), FluTBI (20.8%) and TBF (18.4%) conditioning.

**Table 1:** Patient, donor, and transplant characteristics according to GVHD prophylaxis for all patients.

	PT-Cy + CSA + MMF (*n* = 1528)	PT-Cy + TAC + MMF (*n* = 899)	*P* value
**Age** (years), median (min-max)	55.1 (18.1–75.6)	54.9 (18–82.5)	0.17
**Patient sex**			0.68
Male / female	56.5% / 43.4%	55.6% / 44.4%	
**Karnofsky performance score**	*n* = 1460	*n* = 856	0.56
<90	20.8%	21.8%	
≥90	79.2%	78.2%	
**HCT-CI**	*n* = 1235	*n* = 757	0.72
0–2	76.7%	77.9%	
≥ 3	23.3%	22.1%	
**Cytogenetic risk (ELN 2017)**	*n* = 1344	*n* = 739	0.55
Favorable / Intermediate	6% / 66.9%	5% / 68.7%	
Adverse	27.1%	26.3%	
**MRD pretransplant**	*n* = 700	*n* = 464	0.27
Negative	62.6%	65.7%	
Positive	37.4%	34.3%	
**Donor**			< 0.0001
Haploidentical			
Unrelated	81%	67.4%	
- 10/10 HLA matched	5.8%	16.6%	
- 9/10 HLA matched	8.2%	7.9%	
- HLA not reported	5.0%	8.1%	
**Female donor, male recipient**			0.18
No	80.3%	82.5%	
Yes	19.7%	17.5%	
**Patient CMV status**			0.0002
Negative	24.8%	18.3%	
Positive	75.2%	81.7%	
**Donor CMV status**			0.11
Negative	42.1%	38.8%	
Positive	57.9%	61.3%	
**Cell source**			<0.0001
Bone marrow	31.3%	16.1%	
PBSC	68.7%	83.9%	
**Conditioning**			0.38
MAC	49.8%	47.9%	
RIC	50.2%	52.1%	
**Time diagnosis to HCT** (mo), median (min-max)	5.3 (1–23.9)	5.5 (1.6–23.9)	0.002
**Year of HCT, median (min-max)**	2019 (2010–2021)	2019 (2010–2021)	0.13
**Busulfan**			<0.0001
BuCy	1.5%	1.3%	
BuFlu	19.9%	35.9%	
TBF	56.3%	18.4%	
**TBI**			
FluTBI	13.5%	20.8%	
Other	1.4%	4.0%	
**Melphalan**			
FluMel, FTM	3.1%	8.7%	
**Treosulfan**			
FluTreo	3.4%	8.1%	
Other	0.9%	2.8%	

*PT-Cy* post-transplant cyclophosphamide, *CSA* cyclosporine A, *MMF* mycophenolate mofetil, *TAC* tacrolimus, *HCT-CI* hematopoietic cell transplantation-comorbidity index, *ELN* European LeukemiaNet, *MRD* measurable residual disease, *CMV* cytomegaly virus, *PBSC* peripheral blood stem cells, *MAC* myeloablative conditioning, *RIC* reduced-intensity conditioning, *HCT* hematopoietic cell transplantation, *mo* months, *BuCy* busulfan, cyclophosphamide, *BuFlu* busulfan, fludarabine, *TBF* thiotepa, busulfan, fludarabine, *TBI* total body irradiation, *FluTBI* fludarabine, total body irradiation, *FluMel* fludarabine, melphalan, *FTM* fludarabine, thiotepa, melphalan, *FluTreo* fludarabine, treosulfan.

### Outcome

**Fig. 1:**
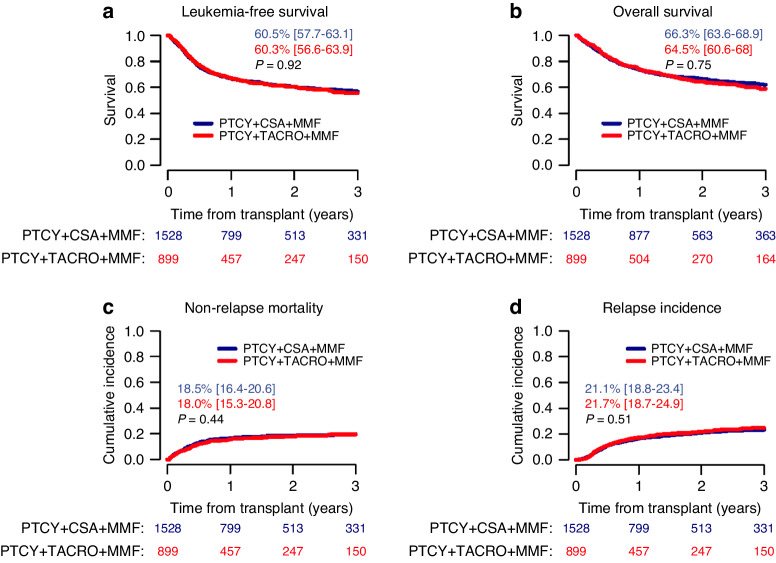
Outcome of patients according to graft-versus-host disease (GVHD) prophylaxis. **a** Leukemia-free Survival; **b** Overall Survival; **c** Non-relapse Mortality; **d** Relapse Incidence.

**Fig. 2:**
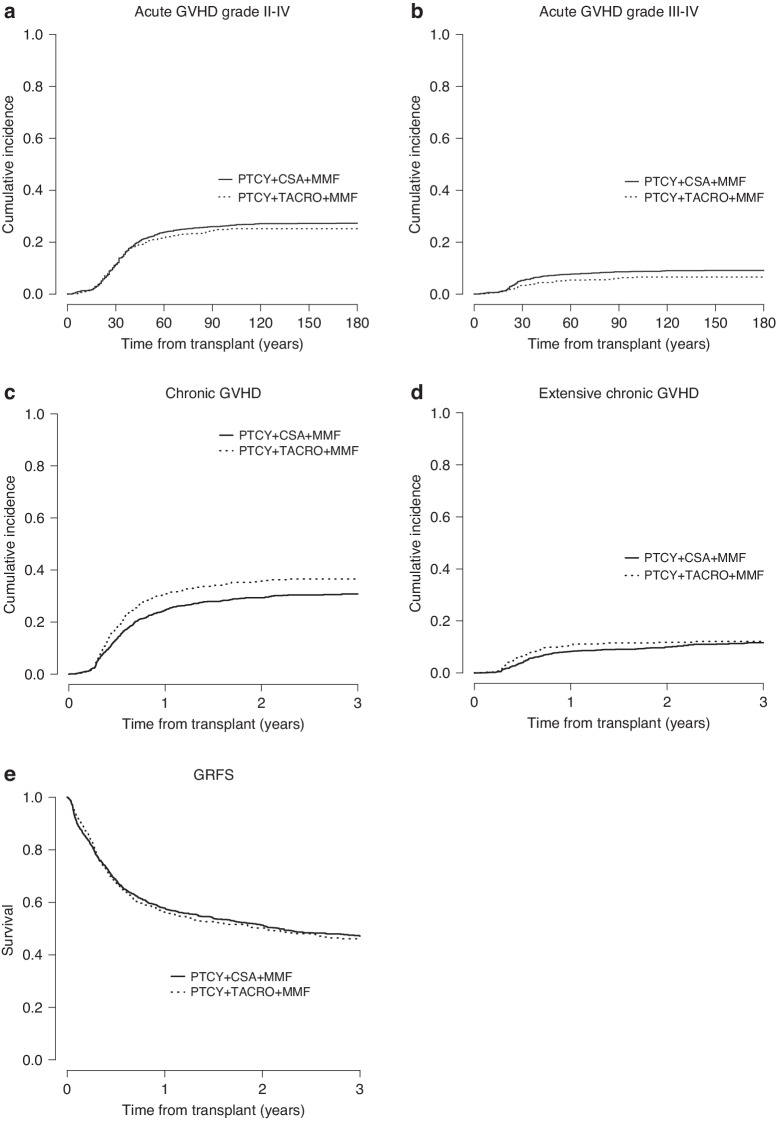
Graft-versus-host disease (GVHD)-based endpoints according to GVHD prophylaxis. **a** Acute GVHD Grade II–IV; **b** Acute GVHD Grade III-IV; **c** Chronic GVHD; **d** Extensive chronic GVHD; **e** Grade III-IV acute GVHD or severe chronic GVHD, relapse-free survival (GRFS).

In univariate analysis, no difference between groups was observed with regard to 2-year LFS and OS (LFS, CSA: 60.5% vs. TAC: 60.3%, *p* = 0.92; OS, 66.3% vs. 64.5%, *p* = 0.75, respectively) as well as NRM (18.5% vs. 18.0%, *p* = 0.44) and CI of relapse (21.1% vs. 21.7%, *p* = 0.51), Fig. 1a–d. Overall, leukemia, infections and GVHD contributed to 43.4%, 27.4% and 12% of deaths, respectively. Graft failure occurred in 3.9% and 5.4% of HCTs with CSA and TAC, respectively, *p* = 0.1. Of note, TAC-based immunosuppression was associated with a lower CI of severe grade III-IV acute GVHD (CSA: 9.1% vs. TAC: 6.6%, *p* = 0.033) but not of grade II-IV acute GVHD (27.3% vs. 25.2%, *p* = 0.34), Fig. 2a, b. The CI of chronic GVHD was higher in the TAC cohort (29.3% vs. 35.7%, *p* = 0.002), whereas the CI of extensive chronic GVHD was not significantly different (9.9% vs. 11.8%, *p* = 0.25), Fig. 2c, d. The choice of CNI did not impact GRFS (51.3% vs. 50.2%, *p* = 0.86), Fig. 2e. After adjusting for confounding factors in a multivariate Cox analysis, the benefit of TAC in reducing severe grade III-IV acute GVHD was confirmed (hazard ratio [HR] 0.63 [95% CI, 0.43–0.93], *p* = 0.02, Table 2), whereas neither chronic GVHD nor other outcome parameters showed a statistically significant difference between CSA and TAC cohorts. Importantly, no interaction was found between GVHD prophylaxis and donor type (data not shown).

**Table 2:** Multivariate analysis on all patients: effect of GVHD prophylaxis on outcomes.

PT-Cy + MMF plus	Relapse	NRM	LFS	OS	GRFS
Tacrolimus vs. Cyclosporine A^a^	1.07 (0.84–1.36)	0.87 (0.67–1.14)	0.95 (0.8–1.13)	0.97 (0.81–1.17)	0.97 (0.82–1.14)
**PT-Cy** + **MMF plus**	**Acute GVHD II–IV**	**Acute GVHD III-IV**	**Chronic GVHD**	**Extensive chronic GVHD**	
Tacrolimus vs. Cyclosporine A^a^	0.84 (0.64–1.1)	**0.63 (0.43–0.93)**	1.24 (0.97–1.58)	1.13 (0.75–1.7)	

^a^All results are given as hazard ratios (95% CI).

The bold value indicates statistical significance, with a *p*-value less than 0.05.

*PT-Cy* post-transplant cyclophosphamide, *MMF* mycophenolate mofetil, *NRM* non-relapse mortality, *LFS* leukemia-free survival, *OS* overall survival, *GRFS* grade III-IV acute GVHD or severe chronic GVHD, relapse-free survival, *GVHD* graft-versus-host disease.

### Multivariate analysis of subgroups according to hematopoietic cell donor

In order to better understand the impact of TAC versus CSA in preventing severe acute GVHD, we additionally applied multivariate analysis to data from patients transplanted from a haploidentical or UD, including parameters distributed differentially between these cohorts (Supplemental Tables 1 and 2) or known to influence outcome. In the UD group, the analysis was restricted to 10/10 (*n* = 238) or 9/10 HLA-matched donors (*n* = 196), excluding 149 of 583 (26%) patients for whom HLA matching was not reported. Our analyses demonstrate a lower CI of severe grade III-IV acute GVHD with TAC solely in the haploidentical donor recipients (HR 0.64 [95% CI, 0.42–0.98], *p* = 0.04) (Table 3), but not in the UD group (HR 0.49 [95% CI, 0.2–1.21], *p* = 0.12) (Table 4). Other outcome parameters were not affected (Tables 3, 4). In the haploidentical, but not unrelated donor group, multivariate analysis also revealed a significantly higher CI of grade II–IV acute GVHD (HR 1.9 [95% CI, 1.45–2.5], *p* < 0.0001), severe grade III-IV acute GVHD (HR 1.79 [95% CI, 1.16–2.75], *p* = 0.008) and chronic GVHD (HR 1.56 [95% CI, 1.2–2.02], *p* < 0.0008) with a peripheral blood stem cell compared to bone marrow graft, respectively.

**Table 3:** Multivariate analysis: effect of GVHD prophylaxis on outcomes - patients with haploidentical donor HCT (*n* = 1844).

PT-Cy + MMF plus	Relapse	NRM	LFS	OS	GRFS
Tacrolimus vs. Cyclosporine A^a^	1.08 (0.86–1.37)	0.85 (0.64–1.12)	0.96 (0.8–1.15)	0.94 (0.77–1.15)	0.96 (0.81–1.15)
**PT-Cy** + **MMF plus**	**Acute GVHD II-IV**	**Acute GVHD III-IV**	**Chronic GVHD**	**Extensive chronic GVHD**	
Tacrolimus vs. Cyclosporine A^a^	0.82 (0.61–1.1)	**0.64 (0.42–0.98)**	1.2 (0.92–1.57)	1.08 (0.7–1.67)	

^a^All results are given as hazard ratios (95% CI).

The bold value indicates statistical significance, with a *p*-value less than 0.05.

*PT-Cy* post-transplant cyclophosphamide, *MMF* mycophenolate mofetil, *NRM* non-relapse mortality, *LFS* leukemia-free survival, *OS* overall survival, *GRFS* grade III-IV acute GVHD or severe chronic GVHD, relapse-free survival, *GVHD* graft-versus-host disease.

**Table 4:** Multivariate analysis: effect of GVHD prophylaxis on outcomes - patients with unrelated donor HCT (10/10 or 9/10 HLA matched).

PT-Cy + MMF plus	Relapse	NRM	LFS	OS	GRFS
Tacrolimus vs. Cyclosporine A^a^	1.09 (0.62–1.9)	1.12 (0.54–2.32)	1.01 (0.67–1.54)	1.23 (0.8–1.88)	1 (0.66–1.5)
**PT-Cy** + **MMF plus**	**Acute GVHD II-IV**	**Acute GVHD III-IV**	**Chronic GVHD**	**Extensive chronic GVHD**	
Tacrolimus vs. Cyclosporine A^a^	1.05 (0.59–1.88)	0.49 (0.2–1.21)	1.35 (0.81–2.25)	1.61 (0.62–4.22)	

^a^All results are given as hazard ratios (95% CI).

*PT-Cy* post-transplant cyclophosphamide, *MMF* mycophenolate mofetil, *NRM* non-relapse mortality, *LFS* leukemia-free survival, *OS* overall survival, *GRFS* grade III-IV acute GVHD or severe chronic GVHD, relapse-free survival, *GVHD* graft-versus-host disease.

## Discussion

GVHD prophylaxis with PT-Cy is increasingly used to transplant patients with AML. While the original Baltimore protocol for haploidentical donor transplants combined PT-Cy with the CNI TAC and MMF, many transplant centers administer CSA instead of TAC, in view of the promising results reported by Raiola et al. [10]. We evaluated whether CSA- and TAC-based GVHD prophylaxis led to similar outcomes in AML patients receiving a haploidentical family donor or UD transplant in CR1. Our study demonstrates similarly favorable OS and GRFS with both TAC and CSA combined with PT-Cy/MMF. TAC was associated with less severe acute GVHD grade III-IV than CSA in haploidentical HCT while no impact on survival and other outcome parameters was observed.

The two randomized comparisons of CSA and TAC involving UD-HCT published to date assessed the combination of these CNIs with MTX and not with PT-CY as examined in our study. In the randomized trial by Nash et al. evaluating UD-HCT only [19], TAC and MTX resulted in significantly less grade II-IV acute GVHD than with CSA and MTX (56% vs. 74%, *p* < 0.001) with a lower severity overall (*p* = 0.005) but no difference in other outcome parameters. Adverse events were similar except for an increased incidence of short-term nephrotoxicity in the TAC group. However, the patient cohort differed substantially from that in our study, with most patients in the latter having chronic myeloid leukemia and only 5% AML in CR1, younger age (median age 35 years), and grafted with bone marrow after MAC exclusively. Similarly, less grade II–IV acute GVHD was observed with TAC versus CSA in combination with either MTX or steroids in a small Japanese randomized trial in which half of the patients received an HCT from a sibling donor [20].

In a retrospective study of the Center for International Blood and Marrow Transplant Research (CIBMTR) assessing the combination of CNI and MMF for MAC UD-HCT in patients with various hematologic malignancies including 50% AML, Hamilton et al. reported less grade III-IV acute GVHD (25% vs. 40%, *p* = 0.001) and 2-year NRM (29% vs. 44%, *p* < 0.01) as well as improved 2-year OS (44% vs. 34%, *p* = 0.05) in patients treated with TAC and MMF (*n* = 424) compared to CSA and MMF (*n* = 68). In contrast to our study, PT-Cy was not used, but antithymocyte globulin (ATG) administered to 34% of patients in each group [21].

In the setting of haploidentical HCT, timing of immunosuppressive therapy in combination with PT-Cy has been shown to influence outcome [22]: For 509 patients with acute leukemia of all stages, an increased probability of LFS (HR 0.58, *p* = 0.2) and refined GRFS (HR 0.62, *p* = 0.3) as well as a reduced risk of relapse (HR 0.49, *p* = 0.3) was reported if PT-Cy was given on days +3 and +5 combined with CSA and MMF from day 0 and +1 (early CSA/MMF), respectively, instead of PT-Cy on days +3 and +4 combined with TAC/MMF from day +5 (late TAC/MMF). In this retrospective study and discrepant with ours, no significant differences in any outcome parameter between late TAC/MMF and late CSA/MMF were observed. Our study considered all schedules of PT-CY/CSA/MMF and was based on a much larger, more homogeneous, and marginally overlapping cohort of 1844 AML patients transplanted from a haploidentical donor, exclusively in CR1. Nevertheless, we cannot exclude a beneficial effect of early CSA/MMF administration.

Interestingly, the higher proportion of mismatched (9/10) UD grafts in the PT-Cy/CSA/MMF group in our study did not translate into inferior outcome compared with fully matched UD-HCT, as could have been expected by the studies of Fleischhauer et al. [23] and Fürst et al. [24], which demonstrated significantly higher overall mortality in mismatched HCT without in vivo T-cell depletion. Thus, our analysis strongly suggests that PT-Cy-based GVHD prophylaxis can overcome the drawback of a mismatched graft in patients with acute leukemia, as also indicated by a recent retrospective analysis [25].

MMF is spontaneously hydrolyzed to its active form MPA, and metabolized further to the inactive hydroxyl-β-glucuronide (MPAG). Substantial enterohepatic cycling of MPA is facilitated by biliary secretion and colonic bacterial deconjugation of MPAG and results in a second peak of plasma MPA [26]. CSA reduces enterohepatic cycling and thereby accelerates MPA clearance by one third compared to TAC [27]. As low total MPA plasma concentrations at steady state have been linked to a higher risk of grade III-IV acute GVHD and NRM in UD-HCT following NMA HCT [28], choice of TAC may be associated with higher MMF plasma levels and better GVHD prevention.

Despite the large and homogeneous patient cohort, our retrospective study did not consider a number of parameters that may be mechanistically important when comparing immunosuppressive potencies of CNIs. For example, plasma MPA levels are not routinely monitored in clinical practice and are subject to many additional parameters, such as: (i) the percentage of patients receiving 45 mg/kg/day, administered t.i.d. up to a maximum of 3 g, which is the recommended dose and schedule for GVHD prophylaxis [29–31]; (ii) the route and duration of administration; and (iii) the serum albumin concentration, which correlates with MPA clearance because MPA is highly bound to serum albumin [27].

Limitations of our study also include unequal distribution of patient characteristics in the haploidentical donor group with respect to the conditioning regimen and hematopoietic cell source. Considering the significantly reduced relapse risk (HR 0.53, *p* = 0.03) and better long-term OS observed with TBF compared to FluBu conditioning irrespective of donor type (62% vs. 48% at 8 years, HR 0.62, *p* = 0.03) [32], a better outcome could have been expected in our CSA cohort in which TBF predominated. Such an effect was not observed in our study in which the predominant combination of FluBu or FluTBI with TAC yielded equivalent survival outcomes in conjunction with superior prevention of severe acute GVHD.

In conclusion, PT-Cy/MMF-based GVHD prophylaxis may result in favorable OS and GRFS in the setting of a haploidentical or UD-HCT, irrespective of the CNI added. In haploidentical HCT, TAC seemed to prevent severe acute GVHD more effectively than CSA without impact on other outcome parameters and may be the preferred combination partner with MMF for patients with AML in CR1.

## Supplementary information

Supplemental Tables

Supplementary Information

## Data Availability

Upon request to the ALWP of the EBMT (Dr Myriam Labopin).
