# Supplementary material for: Tacrolimus versus cyclosporine a combined with post-transplantation cyclophosphamide for AML In first complete remission: a study from the acute leukemia working party (EBMT)
Source: Bone Marrow Transplant. 2024 Jul 3;59(10):1394–401. doi: 10.1038/s41409-024-02331-1 (PMC11452337; doi:10.1038/s41409-024-02331-1)
Supplement: Supplementary file 1 — Supplemental Tables [file 41409_2024_2331_MOESM1_ESM.docx]

Supplemental Table 1: Patient, donor, and transplant characteristics according to GVHD prophylaxis **in unrelated donor HCT**

|  | PT-Cy + CSA + MMF  (n = 290) | PT-Cy + TAC + MMF  (n = 293) | *P* value |
| --- | --- | --- | --- |
| **Age** (years), median (min-max) | 54.1 (18.2-75.6) | 47.8 (18-75.3) | 0.01 |
| **Patient sex**  Male / female | 54.5% / 45.5% | 52.9% / 47.1% | 0.7 |
| **Karnofsky performance score**  <90  ≥90 | n = 278  18%  82% | n = 275  17.8%  82.2% | 0.96 |
| **HCT-CI**  0-2  ≥ 3 | n = 251  72.1%  27.9% | n = 757  81.4%  18.6% | 0.037 |
| **Cytogenetic risk (ELN 2017)**  Favorable / Intermediate  Adverse | n = 263  8.4% / 69.2%  22.4% | n = 215  5.6% / 73%  21.4% | 0.45 |
| **MRD pretransplant**, n (%)  Negative  Positive | n = 151  54.3%  45.7% | n = 127  74.8%  25.2% | <10^-3^ |
| **HLA matches**  10/10  9/10 | n = 214  41.6%  58.4% | n = 220  67.7%  32.3% | <10^-3^ |
| **Female donor, male recipient**  No  Yes | 83.3%  16.7% | 86%  114% | 0.37 |
| **Patient CMV status**  Negative  Positive | 33%  67% | 19.2%  80.8% | <10^-3^ |
| **Donor CMV status**  Negative  Positive | 59.7%  40.3% | 45%  55% | <10^-3^ |
| **Cell source**  Bone marrow  PBSC | 7.9%  92.1% | 2.7%  97.3% | 0.005 |
| **Conditioning**  MAC  RIC | 52.4%  47.6% | 49.8%  50.2% | 0.52 |
| **Time diagnosis to HCT** (mos), median (min-max) | 5.3 (1.4-22.3) | 6.7 (1.6-21.1) | <10^-3^ |
| **Year of HCT, median (min-max)** | 2020 (2010-2021) | 2019 (2010-2021) | <10^-3^ |
| **Conditioning regimen**  **Busulfan**  BuCy  BuFlu  TBF | 1.4%  47.2%  27.6% | 3.8%  51.2%  10.6% |  |
| **TBI**  FluTBI  Other | 7.2%  1.7% | 17.1%  1.0% |  |
| **Melphalan**  FluMel, FTM | 7.2% | 5.8% |  |
| **Treosulfan**  FluTreo | 6.9% | 7.5% |  |
| Other | 0.8% | 3.0% |  |

Supplemental Table 2: Patient, donor, and transplant characteristics according to GVHD prophylaxis **in haploidentical HCT**

|  | PT-Cy + CSA + MMF  (n = 1238) | PT-Cy + TAC + MMF  (n = 606) | *P* value |
| --- | --- | --- | --- |
| **Age** (years), median (min-max) | 55.3 (18.1-75.6) | 56.8 (18.2-82.5) | 0.13 |
| **Patient sex**  Male / female | 56.9% / 43.1% | 56.9% / 43.1% | 0.99 |
| **Karnofsky performance score**  <90  ≥90 | n = 1182  21.5%  78.5% | n = 581  23.8%  76.2% | 0.28 |
| **HCT-CI**  0-2  ≥ 3 | n = 984  77.8%  22.2% | n = 510  76.2%  23.7% | 0.28 |
| **Cytogenetic risk (ELN 2017)**  Favorable / Intermediate  Adverse | n = 984  5.4% / 66.3%  28.4% | n = 335  4.8% / 67%  28.2% | 0.87 |
| **MRD pretransplant**, n (%)  Negative  Positive | n = 549  64.8%  35.2% | n = 337  62.3%  37.7% | 0.45 |
| **Female donor, male recipient**  No  Yes | 79.6%  20.4% | 80.8%  19.2% | 0.55 |
| **Patient CMV status**  Negative  Positive | 22.9%  77.1% | 17.8%  82.2% | 0.012 |
| **Donor CMV status**  Negative  Positive | 38.0%  62.0% | 35.7%  64.3% | 0.35 |
| **Cell source**  Bone marrow  PBSC | 36.8%  63.2% | 22.6%  77.4% | <0.0001 |
| **Conditioning**  MAC  RIC | 49.2%  50.8% | 47%  53% | 0.38 |
| **Time diagnosis to HCT** (mos), median (min-max) | 5.3 (1-23.9) | 5.1 (1.8-23.9) | 0.43 |
| **Year of HCT, median (min-max)** | 2019 (2010-2021) | 2018 (2010-2021) | 0.39 |
| **Conditioning regimen**  **Busulfan**  BuCy  BuFlu  TBF | 1.5%  13.5%  63.1% | 0  28.5%  22.1% |  |
| **TBI**  FluTBI  Other | 15%  1.4% | 22.2%  5.4% |  |
| **Melphalan**  FluMel, FTM | 2.2% | 10.2% |  |
| **Treosulfan**  FluTreo | 2.6% | 8.4% |  |
| Other | 0.7% | 3.2% |  |
