# Supplementary material for: Tacrolimus versus cyclosporine a combined with post-transplantation cyclophosphamide for AML In first complete remission: a study from the acute leukemia working party (EBMT)
Source: Bone Marrow Transplant. 2024 Jul 3;59(10):1394–401. doi: 10.1038/s41409-024-02331-1 (PMC11452337; doi:10.1038/s41409-024-02331-1)
Supplement: Supplementary file 2 — Supplementary Information [file 41409_2024_2331_MOESM2_ESM.docx]

Supplementary Information: Contributing institutions

RM Gorbacheva Research Institute, Pavlov University, Petersburg, Russian Federation; Programme de Transplantation&Therapie Cellulaire, Marseille, France; IRCCS Ospedale Policlinico San Martino, Genova, Italy; Institute of Hematology and Blood Transfusion, Prague, Czech Republic; Universita Cattolica S. Cuore, Rome, Italy; Hospital Gregorio Marañón, Madrid, Spain; Hospital Clínico, Salamanca, Spain; Istituto Clinico Humanitas, Milano, Italy; Leiden University Hospital, Leiden, Netherlands; Turku University Hospital, Turku, Finland; Grande Ospedale Metropolitano Bianchi Melacrino Morelli - Centro Unico Trapianti A. Neri, Reggio Calabria, Italy; Medicana International Hospital Istanbul, Istanbul, Turkey; Hospital U. Marqués de Valdecilla, Santander, Spain; S.S.C.V.D Trapianto di Cellule Staminali, Torino, Italy; CHU Bordeaux, Hopital Haut-Leveque, Pessac, France; University Hospital Eppendorf, Hamburg, Germany; CHU Grenoble Alpes - Université Grenoble Alpes, Grenoble, France; Demiroglu Bilim University Istanbul Florence Nightingale Hospital, Istanbul, Turkey; Medizinische Universitaet Wien, Vienna, Austria; Département d'Oncologie, Service d'Hématologie, Geneva, Switzerland; Anadolu Medical Center Hospital, Kocaeli, Turkey; Saint-Louis Hospital, BMT Unit, Paris, France; Hospital Clinic, Barcelona, Spain; CHU de Lille, Lille, France; ASST GRANDE OSPEDALE METROPOLITANO NIGUARDA, Milano, Italy; Goethe-Universitaet, Frankfurt Main, Germany; Imperial College Hammersmith London, London, United Kingdom; University of Napoli, Napoli, Italy; University Hospital | Basel, Basel, Switzerland; Hospital Ramón y Cajal, Madrid, Spain; Universitaetsklinikum Dresden, Dresden, Germany; Universite Paris IV, Hopital la Pitié-Salpêtrière, Paris, France; European Institute of Oncology, Milano, Italy; Klinikum Grosshadern, Munich, Germany; Evangelismos Hospital, Athens, Greece; Azienda Ospedaliero Universitaria di Udine, Udine, Italy; Centro Trapianti Unico Di CSE Adulti e Pediatrico A. O Brotzu, Cagliari, Italy; H SS. Antonio e Biagio, Alessandria, Italy; Klinikum Augsburg, Augsburg, Germany; AORMN Hospital, Pesaro, Italy; Hospital Sirio-Libanes, Sao Paulo, Brazil; Oslo University Hospital, Rikshospitalet, Oslo, Norway; Ospedale Infantile Regina Margherita, Torino, Italy; Hospital Univ. 12 de Octubre, Madrid, Spain; San Matteo Pavia Transplant Programme, Pavia, Italy; Hopital La Miletrie, Poitiers, France; University Hospital Maastricht, Maastricht, Netherlands; CHU Nantes, Nantes, France; Hannover Medical School, Hannover, Germany; U.O.S.A Centro Trapianti e Terapia Cellulare, Siena, Italy; U.O.D Trapianti di midollo osseo, Rozzano, Italy; IRCCS, Casa Sollievo della Sofferenza, San Giovanni, Italy; Ospedale San Gerardo, Monza, Italy; Baskent University Hospital, Adana, Turkey; Charles University Hospital, Pilsen, Czech Republic; Univ. La Sapienza, Rome, Italy; University Hospital | Essen, Essen, Germany; Dél-pesti Centrumkórház, Budapest, Hungary; Centre Hospitalier Lyon Sud, Lyon, France; Unita Operativa di Ematologia e Trapianto di cellule staminali, Lecce, Italy; Klinikum Frankfurt (Oder) GmbH, Frankfurt Oder, Germany; Cliniques Universitaires St. Luc, Brussels, Belgium; Ospedale S. Camillo-Forlanini, Rome, Italy; Elisabethinen-Hospital, Linz, Austria; CHU Lapeyronie, Montpellier, France; University of Amiens: CHU Amiens, Amiens, France; University Hospital Gasthuisberg, Leuven, Belgium; Azienda Ospedaliera Universitaria Careggi, Firenze, Italy; Fundación Jiménez Díaz, Madrid, Spain; HUCH Comprehensive Cancer Center, Helsinki, Finland; King Hussein Cancer Centre Adult BMT Program, Amman, Jordan; Ospedale La Maddalena - Dpt. Oncologico, Palermo, Italy; Hospital Morales Meseguer, Murcia, Spain; Gazi University Faculty of Medicine, Ankara, Turkey; Hosp. Reina Sofia, Córdoba, Spain; Institut de Cancerologie Lucien Neuwirth, Saint Etienne, France; Hospital San Maurizio, Bolzano, Italy; University of Cologne, Cologne, Germany; Hospital de Gran Canaria Dr Negrin, Las Palmas, Spain; Hospital Universitario Donostia, S Sebastian, Spain; Az. Ospedaliera S. Croce e Carle, Cuneo, Italy; University Medical Center Mainz, Mainz, Germany; Azienda Ospedaliero Universitaria Pisana, Pisa, Italy; University Clinical Centre in Gda?sk, Gdansk, Poland; Centre Henri Becquerel, Rouen, France; Fondazione IRCCS - Ca? Granda, Milano, Italy; Fundeni Clinical Institute, Bucharest, Romania; Clatterbridge Cancer Centre Liverpool, Liverpool, United Kingdom; CHU Nice - Hôpital de l`ARCHET I, Nice, France; AZ Delta, Roeselare, Belgium; A.O.R.N. `SAN.G MOSCATI`, Avellino, Italy; USD Trapianti di Midollo, Adulti, Brescia, Italy; CHU CAEN, Caen, France; Hôpital Henri Mondor, Creteil, France; University Hospital Center Rebro, Zagreb, Croatia; Klinikum Rechts der Isar, Munich, Germany; Universitair Ziekenhuis Brussel, Brussels, Belgium; Philipps Universitaet Marburg, Marburg, Germany; Secretary and Italian National BMT Registry - GITMO, Bergamo, Italy; University Hospital | Linkoeping, Linkoeping, Sweden; University Hospital Erlangen, Erlangen, Germany; CHRU Limoges, Limoges, France; RVI Newcastle, Newcastle, United Kingdom; University Hospitals Bristol and Weston NHSFT, Bristol, United Kingdom; Department of Bone Marrow Transplantation and Oncohematology, Gliwice, Poland; University of Cape Town Faculty of Health Sciences, Cape Town, South Africa; CHRU, Angers, France; Gustave Roussy Cancer Campus, Villejuif, France; Azienda Ospedali Riuniti di Ancona, Ancona, Italy; University of Freiburg, Freiburg, Germany; Antwerp University Hospital (UZA), Antwerp E, Belgium; Hopital Necker Adults, Paris, France; CHU - Institut Universitaire du Cancer Toulouse, Toulouse, France; CHRU NANCY, Vandoeuvre les Nancy, France; Fundació Institut d`Investigació Sanitària Illes Balears ? IdISBa, Palma, Spain; University of Liege, Liege, Belgium; Clinica Puerta de Hierro, Madrid, Spain; Kings College Hospital London, London, United Kingdom; University Regensburg, Regensburg, Germany; University Hospital Ostrava, Ostrava, Czech Republic; University College London Hospital, London, United Kingdom; Hopital Jean Minjoz, Besancon, France; Ospedale Civile, Pescara, Italy; CHU ESTAING, Clermont, France; Klinikum Karlsruhe gGmbH, Karlsruhe, Germany; ZNA, Antwerp, Belgium; University of Heidelberg, Heidelberg, Germany; Addenbrookes Hospital Cambridge, Cambridge, United Kingdom; Cardarelli Hospital, Napoli, Italy; Policlinico G.B. Rossi, Verona, Italy; U.O. Ematologia con Trapianto, Bari, Italy; Centre Hospitalier Universitaire de Rennes, Rennes, France; ICANS - Institut de cancérologie Strasbourg Europe, Strasbourg, France; Institute of Hematology and Transfusion Medicine, Warsaw, Poland; Institut Catalá d`Oncologia Hospital Duran i Reynals, Barcelona, Spain; S. Bortolo Hospital, Vicenza, Italy; Perrino Hospital, Brindisi, Italy; Universitaetsmedizin Mannheim, Mannheim, Germany; Institut Jules Bordet, Brussels, Belgium; Nijmegen Medical Centre, Nijmegen, Netherlands; Research Committee - University of Patras, Patras, Greece; Hospital Clínico de Valencia, Valencia, Spain; Birmingham Centre for Cellular Therapy and Transplant (BCCTT), Birmingham, United Kingdom; Sahlgrenska University Hospital, Goeteborg, Sweden; Hospital Álvaro Cunqueiro - Complejo Hospitalario Universitario de Vigo, Vigo, Spain; King Abdul - Aziz Medical City, Riyadh, Saudi Arabia; University Medical Center Groningen (UMCG), Groningen, Netherlands; George Papanicolaou General Hospital, Thessaloniki, Greece; C.H.R.U de Brest, Brest, France; Nottingham City Hospital, Nottingham, United Kingdom; Umea University Hospital, Umeå, Sweden; Hopital Saint Antoine, Paris, France; King Faisal Specialist Hospital and Research Center, Jeddah, Saudi Arabia; Klinik fuer Innere Medzin III, Ulm, Germany; Bologna University, S.Orsola-Malpighi Hospital, Bologna, Italy; Univ. of Parma, Parma, Italy; LKH - University Hospital Graz, Graz, Austria; Ospedale Nord, Taranto, Italy; Medical Clinic and Policinic 1, Leipzig, Germany; Ospedale Dell'Angelo, Venezia, Italy; A.Z. Sint-Jan, Gent, Belgium; Azienda Ospedaliero Universitaria di Modena Policlinico, Modena, Italy; University Hospital La Fe, Valencia, Spain; Clínica Universitaria de Navarra, Pamplona, Spain; Hospital Universitario Virgen del Rocío, Sevilla, Spain; University of Saarland, Homburg, Germany; Sezione di Ematologia, Perugia, Italy; Medical School University of Salerno, Salerno, Italy; Instituto de Cancerologia S.A, Medellin, Colombia; Mazzoni Hospital, Ascoli Piceno, Italy; Rigshospitalet, Herlev, Denmark; University Hospital, Zürich, Switzerland; Hopital Bretonneau, Tours, France; Hospital Universitario Virgen de la Arrixaca, Murcia, Spain; Martin-Luther-Universitaet Halle-Wittenberg, Halle, Germany; Birmingham Centre for Cellular Therapy and Transplant (BCCTT), Stoke, United Kingdom; King Faisal Specialist Hospital & Research Centre, Riyadh, Saudi Arabia; HELIOS Klinikum Berlin-Buch, Berlin, Germany; Hospital Regional de Málaga, Malaga, Spain; Hospital Vall d`Hebron, Barcelona, Spain; Ankara University Faculty of Medicine, Ankara, Turkey; Arcispedale S. Maria Nuova, Reggio E, Italy; Centre Pierre et Marie Curie, Alger, Algeria; Hospital Universitario La Paz, Madrid, Spain; Sheffield Royal Hallamshire, Sheffield, United Kingdom; Ospedale San Raffaele s.r.l., Rome, Italy; FCHBE, St. Petersburg, Russia; Universitaet Bonn, Bonn, Germany; Robert_Bosch_Krankenhaus, Stuttgart, Germany; Tel Aviv Sourasky Medical Center, Tel Aviv, Israel; Hospital Guglielmo da Saliceto, Piacenza, Italy; Karolinska University Hospital, Stockholm, Sweden; Royal Marsden Hospital, London, United Kingdom; Universitaet Tuebingen, Tuebingen, Germany; Hospital de la Princesa, Madrid, Spain; Academisch Ziekenhuis bij de Universiteit, Amsterdam, Netherlands; St James University Hospital Leeds, Leeds, United Kingdom; Hadassah University Hospital, Jerusalem, Israel; Hospital Santa Creu i Sant Pau, Barcelona, Spain; University Hospital | Uppsala, Uppsala, Sweden; ASCTR - Austrian Stem Cell Transplantation Registry, Innsbruck, Austria; Cardiff University Hospital of Wales & Swansea, Cardiff, United Kingdom; American University of Beirut Medical Center, Beirut, Lebanon; Heinrich Heine Universitaet, Duesseldorf, Germany; Singapore General Hospital, Singapore, Singapore; King Fahad Specialist Hospital, Dammam, Saudi Arabia; National Center for Cancer Care & Research, Doha, Qatar; ALBERTS CELLULAR THERAPY, Pretoria, South Africa; Hospital Univ. Virgen de las Nieves, Granada, Spain; Hospital Universitario de Navarra, Pamplona, Spain; ICO-Hospital Universitari Germans Trias i Pujol, Badalona, Spain; Klinikum Nuernberg, Wuerzburg, Germany; Clinical Hospital Merkur, Zagreb, Croatia; Vilnius University Hospital Santaros Klinikos, Vilnius, Lithuania; University of Debrecen Clinical Center, Debrecen, Hungary; Hôpital  D'Instruction des Armées (HIA) PERCY, Clamart, France; Charles University Hospital, Hradec, Czech Republic; Ghent University Hospital, Gent, Belgium; Istanbul Tip Fakultesi, Istanbul, Turkey; Azienda Ospedaliero Universitaria, Bologna, Italy; Ospedale Policlinico, Catania, Italy; FOSCAL Internacional of the FOSUNAB Foundation, Floridablanca, Colombia; Meyer University Children Hospital, Firenze, Italy
